# Investigating the relationship between *UMODL1* gene polymorphisms and high myopia: a case–control study in Chinese

**DOI:** 10.1186/1471-2350-13-64

**Published:** 2012-08-02

**Authors:** Miao-miao Zhu, Maurice KH Yap, Daniel WH Ho, Wai Yan Fung, Po Wah Ng, Yang-shun Gu, Shea Ping Yip

**Affiliations:** 1 Department of Ophthalmology, The First Affiliated Hospital, Zhejiang University School of Medicine, Hangzhou, China; 2 Department of Health Technology and Informatics, The Hong Kong Polytechnic University, Hong Kong SAR, China; 3 Centre for Myopia Research, School of Optometry, The Hong Kong Polytechnic University, Hong Kong SAR, China

**Keywords:** High myopia, *UMODL1*, Single nucleotide polymorphism, Association study, Secondary phenotype

## Abstract

**Background:**

The *UMODL1* gene was found to be associated with high myopia in Japanese. This study aimed to investigate this gene for association with high myopia in Chinese.

**Methods:**

Two groups of unrelated Han Chinese from Hong Kong were recruited using the same criteria: Sample Set 1 comprising 356 controls (spherical equivalent, SE, within ±1 diopter or D) and 356 cases (SE ≤ −8D), and Sample Set 2 comprising 394 controls and 526 cases. Fifty-nine tag single nucleotide polymorphisms (SNPs) were selected and genotyped for Sample Set 1. Four SNPs were followed up with Sample Set 2. Both single-marker and haplotype analyses were performed with cases defined by different SE thresholds. Secondary phenotypes were also analyzed for association with genotypes.

**Results:**

Data filtering left 57 SNPs for analysis. Single-marker analysis did not reveal any significant differences between cases and controls in the initial study. However, haplotype GCT for markers rs220168-rs220170-rs11911271 showed marginal significance (empirical *P* = 0.076; SE ≤ −12D for cases), but could not be replicated in the follow-up study. In contrast, non-synonymous SNP rs3819142 was associated with high myopia (SE ≤ −10D) in the follow-up study, but could not be confirmed using Sample Set 1. The SNP rs2839471, positive in the original Japanese study, gave negative results in all our analyses. Exploratory analysis of secondary phenotypes indicated that allele C of rs220120 was associated with anterior chamber depth (adjusted *P* = 0.0460).

**Conclusions:**

Common *UMODL1* polymorphisms were unlikely to be important in the genetic susceptibility to high myopia in Han Chinese.

## Background

Myopia is the commonest eye defect worldwide. Myopic eyes focus the image of a distant object in front of the retina when accommodation is relaxed. High myopia is often defined as a refractive error of −6.0 diopters (D) or worse, and severely myopic eyes are particularly vulnerable to many ocular pathologies [1]. The increasing prevalence and decreasing earlier age-of-onset of myopia make it a priority in many populations [2]. The prevalence of myopia is much higher in Asian populations (~70%) than in Caucasian populations (15%-27%) [2,3].

Myopia is a complex disease caused by both genetic and environmental factors plus their interactions although the exact etiology is still unclear [4,5]. High heritability observed in many twin and family studies provides convincing evidence that genetic factors play a strong role in the development of myopia [6,7]. Genetic association studies are more powerful than linkage analysis in detecting genes with relatively small effects in complex diseases [5]. While genome-wide association studies are getting more popular, the cost is still prohibitive for many researchers. Candidate gene studies remain important for hunting predisposing factors for complex diseases.

Recently, a Japanese group performed a genome-wide case–control association analysis of high myopia with ~27,000 microsatellite markers and followed up a positive marker (D21S0083i on chromosome 21q22.3) with 39 single nucleotide polymorphisms (SNPs) in a case–control study [8]. The SNP rs2839471 was found to be associated with high myopia even after correction for multiple comparisons by Bonferroni procedure (*P* = 0.00027 and corrected *P* = 0.01). The uromodulin-like 1 (*UMODL1*) gene harboring rs2839471 was suggested as a new susceptibility gene for high myopia.

The *UMODL1* gene (GeneID: 89766) is a protein-coding gene located at chromosome 21q22.3, spans a genomic region of ~80 kb and consists of 23 exons (http://www.ncbi.nlm.nih.gov/). It encodes several transcripts via alternative splicing, among which *UMODL1S* and *UMODL1L* are the two major isoforms that have been better characterized so far. Both isoforms contain multiple domains such as whey acidic protein, calcium-binding EGF-like, fibronectin type III, SEA (sea urchin sperm protein, enterokinase, and agrin), and zona pellucida domains [9]. These domains are typically found in various combinations in extracellular matrix (ECM) proteins, indicating that the UMODL1 protein may be secreted and associated with ECM proteins involved in cell/cell and cell/ECM adhesion and in cell migration [10]. The gene is expressed at low level in the eye [11].

The present study aimed to systematically investigate the *UMODL1* gene for association with high myopia in a Han Chinese population. We performed with a Sample Set 1 an initial case–control study to identify putatively positive SNPs or haplotypes and then replicated the “positive” results by an independent Sample Set 2.

## Methods

### Subjects and DNA samples

Two case–control sample sets were used in this study and collected using the same entry criteria as described previously [12]. Each study subject received a complete ocular examination. Spherical equivalent (SE) within ±1.0 D categorized a subject as an emmetropic control while SE of −8.00 D or less defined a subject as a case with high myopia. The study was approved by the Human Subjects Ethics Subcommittee of the Hong Kong Polytechnic University, and adhered to the tenets of the Declaration of Helsinki. Written informed consents were obtained from all subjects. DNA was extracted from whole blood with a modified salt precipitation method [12] or FlexiGene DNA Kit (Qiagen, Hilden, Germany) according to the manufacturer’s instructions.

### Selection of tag SNPs

In the initial study, 58 tag SNPs were selected from an 86-kb genomic region comprising the *UMODL1* gene and its 3-kb flanking regions (both upstream and downstream) by Haploview [13]. The selection was based on the Han Chinese data from the International HapMap Project (release 23a, phase II; http://www.hapmap.org/) [14] with the following criteria: pairwise tagging algorithm, r^2^ ≥ 0.8, minor allele frequency (MAF) >0.2 and forced inclusion of SNPs that had been tested in the Japanese study. A non-synonymous SNP (rs3819142) was added at a later stage and genotyped for both sample sets because it was in moderate linkage disequilibrium (LD; r^2^ =0.6) with rs220170, which was one of the SNPs constituting a putatively positive haplotype from the initial study.

### Genotyping of SNPs

Three different methods were used to genotype these SNPs: mass spectrometry of multiplexed primer-extended products [15] (MassArray iPLEX; Sequenom, San Diego, CA), restriction fragment length polymorphism (RFLP), and denaturing high performance liquid chromatography (DHPLC) of primer-extended products [16].

In the initial study, 56 SNPs were grouped together with SNPs of other on-going studies and genotyped by a mass spectrometric method according to the manufacturer’s instructions (Sequenom), as reported previously by our group [17-19]. Sequences of primers for PCR and primer extension (PE) are shown in Additional file 1. Two SNPs (rs220140 and rs220173) were genotyped by RFLP because they could not be grouped together with other SNPs for genotyping by the iPLEX assays. Polymerase chain reaction (PCR) was performed in a 10-μl reaction mixture containing 10 ng of DNA template, 0.3 μM or 0.5 μM of each primer (Additional file 2), 0.2 mM of each dNTP and 0.2 U of HotStar*Taq* Plus DNA Polymerase (Qiagen, Hilden, Germany) in 1× PCR buffer (with 1.5 mM MgCl_2_, Qiagen). Amplification was performed in a 96-well PCR machine (GeneAmp PCR system 9700; Applied Biosystems, Foster City, CA). The PCR program was as follows: initial denaturation at 95°C for 5 min, followed by 35 cycles of denaturation at 95°C for 30 seconds, annealing at 60°C for 30 seconds and elongation at 72°C for 30 seconds, plus a final extension at 72°C for 5 minutes. PCR products were digested overnight with a restriction enzyme (MBI Fermentas, Vilnius, Lithuania; Additional file 2) according to the manufacturer’s instructions. Digested products were pre-stained for 15 minutes with SYBR Green I (Invitrogen, Carlsbad, CA) and then separated in 8% 102-well horizontal polyacrylamide gels prepared using a home-made casting cassette.

In the follow-up study, four SNPs were genotyped. Of these, rs220168, rs220170 and rs3819142 were first genotyped by the mass spectrometric method (MassArray iPLEX). Samples that could not be grouped together and genotyped within a SpectroCHIP (for 360 samples; Sequenom) were genotyped by RFLP. Either way, the methods were as described above (Additional file 1 and Additional file 2). On the other hand, rs11911271 was genotyped by PE coupled with DHPLC [16]. The PCR templates were generated using the same conditions as mentioned above and purified by shrimp alkaline phosphatase and exonuclease I (New England Biolabs, Beverly, MA). PE reactions were performed in a 20-μl reaction mixture containing 10 μl of purified PCR products, 1.5 μM of extension primer (Additional file 2), 50 μM of ddCTP/ddTTP and 1 unit of Therminator (New England Biolabs, Beverly, MA) in a 1× reaction buffer supplied by the manufacturer. Thermocycling was performed with an initial denaturation at 96°C for 1 minute, followed by 55 cycles of 96°C for 10 seconds, 43°C for 15 seconds and 60°C for 1 minute. PE products were analyzed by DHPLC (WAVE Nucleic Acid Fragment Analysis System; Transgenomic, Omaha, NE) as described previously [20] with 20% starting buffer B concentration.

### Statistical analysis

Ocular data were analyzed using the SPSS package (version 11.5; SPSS Inc.). Subjects were classified as affected (cases) or unaffected (controls). High myopia was analyzed as a dichotomous trait and a quantitative trait separately. Subset analysis was also performed by defining cases with increasingly restrictive thresholds of refractive error in order to fully explore the genotype data (Sample Sets 1 and 2). Hardy-Weinberg equilibrium (HWE) was tested for controls and cases separately by Fisher’s exact test executed within Plink (version 1.07) [21]. SNPs that were not in HWE were excluded from subsequent association analysis. Haplotype blocks were constructed by Haploview with an algorithm known as the solid spine of linkage disequilibrium (SSLD) [13]. Single-marker and haplotype analyses were performed with three packages: Haploview, Plink and Beagle (ver. 3.0) [22-24]. Multiple testing was corrected by 10,000 permutations to generate empirical *P* values (*P*_emp_). Localized haplotype clusters that were found significantly associated with high myopia by Beagle were further analyzed using the cluster2haps program to identify the haplotypes and SNPs defining the clusters [24].

The SPREG program [25] was used to analyze secondary phenotypes because the case–control subjects were not collected from a random population. SPREG implements *valid* and efficient statistical methods for analyzing secondary phenotypes collected in case–control association studies. It used a modified linear regression method to analyze secondary phenotypes including axial length (AXL), anterior chamber depth (ACD), lens thickness (LT) and corneal power (CP). False discovery rate (FDR) was used to correct for multiple comparisons within a given secondary phenotype [26].

## Results

### Analysis of ocular data

The initial study recruited 712 unrelated Southern Han Chinese subjects (Sample Set 1) including 356 controls and 356 cases defined by SE ≤ -8.0 D (Table 1). SNPs constituting positive haplotypes from the initial study were replicated using a second sample set (Sample Set 2). Sample Set 2 consisted of 920 unrelated Han Chinese subjects with 394 controls and 526 cases defined by SE ≤ -8.0 D (Table 1). There were fewer females in the control group than in the case group: 56.2% versus 69.7% for Sample Set 1 (*P* = 0.0027), and 59.1% versus 67.8% for Sample Set 2 (*P* = 0.0093). The control subjects were on average younger than the case subjects in Sample Set 1 (26.0 versus 28.9 years, P < 0.0001) while both subject groups were of similar age in Sample Set 2 (32.9 versus 32.6 years, *P* = 0.5632). For both sample sets, the controls and the cases had very similar values for anterior chamber depth, lens thickness and corneal power.

**Table 1:** Characteristics of study subjects for sample Set1 and Set 2^*^

	**Sample Set 1**				**Sample Set 2**			
	**Controls**	**Cases**			**Controls**	**Cases**		
Characteristic	± 1 D	≤ -8 D	≤ -10 D	≤ -12 D	± 1 D	≤ -8 D	≤ -10 D	≤ -12 D
Total no.	356	356	206	93	394	526	269	116
Females, no. (%)	200 (56.2)	248 (69.7)	144 (69.9)	67 (72.0)	233 (59.1)	356 (67.8)	194 (72.1)	84 (72.4)
Age, mean (SD), y	26.0 (7.1)	28.9 (7.8)	29.6 (7.8)	31.3 (8.3)	32.9 (9.8)	32.6 (8.9)	32.6 (8.6)	32.6 (9.0)
Spherical equivalent, mean (SD), D	0.01 (0.46)	-10.65 (2.64)	-11.98 (2.73)	-13.76 (3.15)	0.13 (0.54)	-10.29 (2.31)	-11.59 (2.37)	-13.50 (2.34)
Axial length, mean (SD), mm	23.83 (0.82)	27.81 (1.17)	28.19 (1.17)	28.70 (1.23)	23.69 (0.82)	27.60 (1.21)	28.04 (1.24)	28.68 (1.34)
Anterior chamber depth, mean (SD), mm	3.57 (0.34)	3.67 (0.35)	3.66 (0.36)	3.58 (0.37)	3.20 (0.41)	3.37 (0.39)	3.33 (0.40)	3.33 (0.43)
Lens thickness, mean (SD), mm	3.98 (0.48)	4.06 (0.55)	4.08 (0.56)	4.10 (0.50)	4.30 (0.56)	4.22 (0.53)	4.24 (0.54)	4.26 (0.61)
Corneal power, mean (SD), D	43.54 (1.50)	44.50 (1.40)	44.64 (1.38)	44.87 (1.40)	44.04 (1.49)	44.76 (1.47)	44.83 (1.49)	44.99 (1.45)

* The table shows the data for the right eyes only.

Table 1 also shows the summary of cases defined by different thresholds of SE. For Sample Set 1, the number of cases reduced from 356 to 206 to 93 when the threshold SE changed from −8 D to −10 D to −12 D. The mean SE became more negative (from −10.65 D to −11.98 D to −13.76 D), and the axial length longer (from 27.81 mm to 28.19 mm to 28.70 mm) with increasingly stringent thresholds for cases. Similar trends of changes were observed for Sample Set 2 (Table 1).

### Initial association study

The 58 tag SNPs selected on the basis of the Han Chinese data from the HapMap database could capture the genetic information for a total of 125 SNPs in the indicated region (86.0 kb) at a mean r^2^ of 0.971. The non-synonymous SNP rs3819142, which involves an amino acid change (asparagine to histidine), was added as has been explained above. Of the 59 SNPs genotyped, one SNP (rs1571737) was removed because it failed to pass the minimum 80% call rate for the MassARRAY iPLEX assay. Another SNP (rs220279) was also removed because the genotypes were not in HWE in the control group (*P* < 0.001). All the remaining 57 SNPs were in HWE at a threshold *P* value of 0.001 in both controls and cases. These 57 SNPs were designated as S1 to S57 in a sequential order from the 5’ end to the 3’ end of the sense strand of the *UMODL1* gene for the sake of easy referencing (Table 2). Based on the SSLD algorithm of Haploview, 16 haplotype blocks were constructed (Additional file 3). The overall LD among the 57 SNPs under analysis was quite weak.

**Table 2:** **Summary statistics of* UMODL1 *SNPs in initial study (cases defined as spherical equivalent ≤ −8.0 diopters)**

			**Allele†**		**Genotype counts (11/12/22)**		**Minor allele freq**		**Association test (best result)‡**		
SNP*		Location*	**1**	**2**	**Controls**	**Cases**	**Controls**	**Cases**	***P***_**asym**_	**Model**	***P***_**emp**_
rs220260	S1	Intergenic	C	G	149/157/44	155/160/36	0.3500	0.3343	0.3630	Recessive	1
rs220262	S2	Intergenic	A	T	174/148/29	173/157/22	0.2934	0.2855	0.3038	Recessive	1
rs220263	S3	Intergenic	A	G	106/159/83	85/179/86	0.4670	0.4986	0.0673	Dominant	0.9882
rs220265	S4	Intergenic	C	A	127/155/62	106/181/65	0.4055	0.4418	0.0572	Dominant	0.9763
rs12627387	S5	Intergenic	G	A	100/166/86	104/184/66	0.4801	0.4463	0.0614	Recessive	0.9829
rs13340012	S6	Intergenic	A	C	211/117/22	201/134/14	0.2300	0.2321	0.1738	Recessive	1
rs220271	S7	Intron	C	T	128/164/61	135/175/45	0.4051	0.3732	0.0860	Recessive	0.9955
rs220276	S8	Intron	G	A	118/168/66	129/168/52	0.4261	0.3897	0.1650	Allelic	1
rs220278	S9	Intron	G	A	143/157/53	159/153/42	0.3725	0.3347	0.1374	Allelic	0.9997
rs220281	S10	Intron	G	A	157/151/44	149/169/38	0.3395	0.3441	0.4408	Genotypic	1
rs220282	S11	Intron	G	A	139/160/55	126/160/64	0.3814	0.4114	0.2487	Allelic	1
rs220285	S12	Intron	C	G	252/69/11	269/78/7	0.1370	0.1299	0.2740	Recessive	1
rs220298	S13	Intron	G	A	156/159/39	181/144/31	0.3347	0.2893	0.0647	Allelic	0.9867
rs220299	S14	Intron	T	C	156/152/46	141/168/43	0.3446	0.3608	0.2804	Dominant	1
rs8133951	S15	Intron	G	A	126/151/51	135/159/48	0.3857	0.3728	0.5810	Recessive	1
rs749020	S16	Intron	G	A	112/166/76	112/166/75	0.4492	0.4476	0.9425	Recessive	1
rs220301	S17	Intron	G	C	136/156/60	134/166/50	0.3920	0.3800	0.3145	Recessive	1
rs2839466	S18	Intron	G	A	136/165/48	147/160/43	0.3739	0.3514	0.3803	Additive	1
rs220308	S19	Intron	T	G	105/169/80	111/168/74	0.4647	0.4476	0.5187	Allelic	1
rs220109	S20	Exon (syn)	C	T	112/153/83	114/167/72	0.4583	0.4405	0.2706	Recessive	1
rs220110	S21	Intron	C	A	159/141/52	149/154/46	0.3480	0.3524	0.5088	Dominant	1
rs12626854	S22	Intron	T	C	156/156/41	149/156/40	0.3371	0.3420	0.7891	Dominant	1
rs220120	S23	Intron	G	C	165/150/37	180/136/34	0.3182	0.2914	0.2276	Dominant	1
rs220131	S24	Intron	C	T	86/177/79	97/171/83	0.4898	0.4801	0.4574	Dominant	1
rs220136	S25	Intron	C	A	90/185/76	92/169/87	0.4801	0.4928	0.2953	Recessive	1
rs11701944	S26	Intron	A	G	144/152/48	133/169/46	0.3605	0.3750	0.3282	Dominant	1
rs220140	S27	Intron	C	G	161/156/38	164/161/31	0.3268	0.3132	0.3686	Recessive	1
rs220143	S28	Intron	G	A	177/140/35	183/143/23	0.2983	0.2708	0.1071	Recessive	0.9990
rs2839468	S29	Intron	A	C	111/161/82	100/179/72	0.4590	0.4601	0.3391	Genotypic	1
rs220145	S30	Intron	G	A	224/105/18	204/135/17	0.2032	0.2374	**0.0489**	Dominant	0.9569
rs13047454	S31	Intron	A	G	183/138/34	175/160/19	0.2901	0.2797	**0.0331**	Recessive	0.8971
rs220148	S32	Intron	A	C	167/146/39	182/140/28	0.3182	0.2800	0.1182	Allelic	0.9995
rs220149	S33	Intron	T	G	96/165/86	96/185/71	0.4856	0.4645	0.1439	Recessive	0.9999
rs220153	S34	Intron	G	A	212/108/27	199/125/23	0.2334	0.2464	0.3153	Dominant	1
rs220154	S35	Intron	C	T	109/163/77	109/178/64	0.4542	0.4359	0.2066	Recessive	1
rs220155	S36	Intron	C	T	136/144/58	125/162/55	0.3846	0.3977	0.3229	Dominant	1
rs11910495	S37	Intron	A	G	256/90/8	250/92/9	0.1497	0.1567	0.7161	Allelic	1
rs13051533	S38	Intron	G	A	169/136/40	163/147/34	0.3130	0.3125	0.4684	Recessive	1
rs220157	S39	Intron	C	T	126/156/64	121/175/47	0.4104	0.3921	0.0870	Recessive	0.9957
rs9984766	S40	Intron	G	A	226/86/21	204/98/16	0.1922	0.2044	0.3168	Dominant	1
rs220158	S41	Exon (syn)	C	T	214/117/23	225/104/24	0.2302	0.2153	0.3677	Dominant	1
rs220159	S42	Exon (ns)	G	A	136/153/64	120/177/54	0.3980	0.4060	0.1664	Genotypic	1
rs2839470	S43	Intron	C	T	123/163/57	125/168/55	0.4038	0.3994	0.7717	Recessive	1
rs4920063	S44	Intron	G	A	155/139/47	133/184/36	0.3416	0.3626	**0.0100**	Genotypic	0.9219
rs220161	S45	Intron	C	G	92/158/83	106/171/67	0.4865	0.4433	0.0879	Recessive	0.9963
rs2839471	S46	Intron	T	C	98/178/76	97/187/70	0.4688	0.4619	0.5512	Recessive	1
rs220168	S47	Intron	A	G	217/113/21	198/132/21	0.2208	0.2479	0.1447	Dominant	0.9999
rs220170	S48	Intron	C	T	212/123/19	199/130/23	0.2274	0.2500	0.3192	Allelic	1
rs11911271	S49	Intron	T	C	176/148/27	201/122/31	0.2877	0.2599	0.0773	Dominant	0.9929
rs220171	S50	Intron	C	T	158/163/30	185/137/30	0.3177	0.2798	**0.0455**	Dominant	0.9477
rs220172	S51	Intron	G	A	171/137/34	160/139/35	0.2997	0.3129	0.5858	Dominant	1
rs220173	S52	Intron	C	G	88/183/84	97/180/79	0.4944	0.4747	0.4535	Additive	1
rs3819141	S53	Exon (syn)	T	A	128/163/62	120/160/73	0.4065	0.4334	0.2925	Recessive	1
rs3819142	S54	Exon (ns)	A	C	206/130/19	206/130/19	0.2366	0.2514	0.5125	Additive	1
rs220179	S55	Intron	C	T	175/146/27	175/146/27	0.2874	0.2963	0.7097	Additive	1
rs915840	S56	Intron	T	C	212/122/21	212/122/21	0.2310	0.2486	0.3025	Dominant	1
rs220181	S57	Intron	G	A	183/140/15	183/140/15	0.2515	0.2536	0.2039	Recessive	1

*SNPs are listed down the column in sequential order from the 5’ end to the 3’ end of the sense strand of the *UMODL1* gene. They are also designated as S1 to S57 for the sake of easy referencing. SNPs located in exons are either synonymous (syn) or non-synonymous (ns).

†1: major allele; 2: minor allele.

‡Single-marker analysis is performed with Plink. Asymptotic *P* values (*P*_asym_) are obtained by chi-square test, and empirical *P* values (*P*_emp_) obtained by 10,000 permutations for correcting multiple comparisons. The genetic models tested for each SNP are allelic, genotypic, additive (tested by trend test), dominant and recessive. Here, only the best result and the corresponding genetic model are shown for each SNP.

Table 2 summarizes the distribution of genotypes, minor allele frequencies and the results of single-marker association analysis performed with Plink. In particular, no association between high myopia and any SNP was detected under any of the five genetic models tested (allelic, genotypic, additive, dominant and recessive) after correcting for multiple comparisons. The conclusion remained the same even if sex and age were added as covariates for adjustment to avoid their potential confounding effects. Even when the threshold SE for defining cases was more restrictive at −10 D or −12 D, none of the 57 SNPs gave a significant difference between cases and controls after 10,000 permutations. The results were consistent with those generated by the Beagle package (data not shown). The SNP (rs2839471 or S46) that was positive in the Japanese study [8] was found negative in all our analyses.

Haplotype analysis still did not show any significant differences between cases and controls when it was conducted using either LD-block-based model by Haploview or a sliding window strategy by Plink (data not shown in either setting). The same conclusion was drawn when sex and age were added as covariates in the analysis by Plink. However, one haplotype cluster defined by the 0.G allele of rs220168 (S47) showed a marginally significant result (*P*_emp_ = 0.0763 *only* for cases with SE ≤ -12 D) when tested with the Beagle program by a localized haplotype-cluster model and corrected for multiple testing by 10,000 permutations. For rs220168 (S47), the designation “0.G” represented the G allele of the marker (rs220168) at node “0” (zero) in a direct acyclic graph used by Beagle to represent localized haplotype clusters [22,23]. The SNP rs220168 (S47) was in the same LD block as the Japanese positive SNP rs2839471 (S46) (Additional file 3), and the two SNPs are 115 bp apart. Therefore, we further identified the haplotypes that were present in this cluster with the cluster2haps program. The haplotype GCT (211 in the 1–2 or major-minor format) for markers rs220168-rs220170-rs11911271 (S47-S48-S49) showed the most significant association: *P* = 0.0015 with an odds ratio (OR) of 3.78 when compared with all other haplotypes (Table 3). According to the HapMap data, the non-synonymous SNP rs3819142 (S54) was in moderate LD (r^2^ =0.6) with rs220170 (S48). It might have some functional consequences leading to high myopia. Therefore, these SNPs were further tested in a follow-up study using Sample Set 2: rs220168 (S47), rs220170 (S48), rs11911271 (S49) and rs3819142 (S54).

**Table 3:** Identification of haplotypes in localized haplotype clusters that are associated with high myopia

Markers & haplotypes*	**Haplotype counts**		**Haplotype being considered**		**All other haplotypes**		**Fisher's exact test**	
	**Total no.†**	**no. in cluster (%)**	**Controls†**	**Cases†**	**Controls**	**Cases**	***P*value‡**	**OR (95% CI) §**
**Sample Set 1 for cases defined by SE ≤-12 diopters Marker = rs220168 (S47), Cluster = 0.G**								
Markers: S47								
G (2)	202	28 (13.9%)	155	47	557	135	0.2744	1.25 (0.83 - 1.85)
Markers: S47-S48								
GC (21)	35	26 (74.3%)	22	13	690	169	0.0176	2.41 (1.09 - 5.12)
GT (22)	167	2 (1.2%)	133	34	579	148	1.0000	1.00 (0.63 - 1.54)
Markers: S47-S48-S49								
GCT (211)	25	25 (100.0%)	13	12	699	170	**0.0015**	3.78 (1.54 - 9.19)
GCC (212)	10	1 (10.0%)	9	1	703	181	0.6965	0.43 (0.01 - 3.15)
GTC (222)	23	2 (8.7%)	17	6	695	176	0.4411	0.39 (0.44 - 3.77)
Markers: S47-S48-S49-S50								
GCTC (2112)	13	13 (100.0%)	8	5	704	177	0.1546	2.48 (0.63 - 8.73)
GCTT (2111)	12	12 (100.0%)	5	7	707	175	0.0041	5.64 (1.52 - 22.82)
GCCC (2122)	10	1 (10.0%)	9	1	703	181	0.6965	0.43 (0.01 - 3.15)
GTCC (2222)	3	2 (66.7%)	1	2	711	180	0.1071	7.87 (0.41 – 465.3)
**Sample Set 2 for cases defined by SE ≤ -10 diopters Marker = rs3819142 (S54), Cluster = 0.C**								
Markers: S54								
C (2)	290	222 (76.6%)	193	97	595	441	**0.0055**	0.68 (0.51 - 0.90)
Markers: S49-S54								
TC (12)	253	214 (84.6%)	166	87	622	451	0.0274	0.72 (0.54 - 0.97)
CC (22)	37	8 (21.6%)	27	10	761	528	0.0926	0.43 (0.23 - 1.15)

*Markers are rs220168 (S47), rs220170 (S48), rs11911271 (S49), rs220171 (S50) and rs3819142 (S54) along the 5’ end to the 3’ end direction of the sense strand of the *UMODL1* gene. A localized haplotype cluster is indicated in the format of “(node number).(allele of the marker being considered)”. For example, “Marker = rs220168 (S47), Cluster = 0.G” refers to the G allele of the marker S47 at node “0” (zero). The allele of each marker is shown in both the ACGT and the 1–2 formats (1 being the major and 2 the minor allele). For each localized haplotype cluster found to be associated with high myopia by Beagle, the table here shows the haplotypes for an increasing number of markers until the haplotypes account for all or almost all of the association signals for the cluster and give the lowest *P* values (in **boldface**) for the Fisher’s exact test. Then, haplotypes are shown for one extra window with one additional marker just to show that one additional marker does not provide additional information and the *P* values for Fisher’s exact test become less impressive (i.e. larger).

†For a particular haplotype under consideration, the total count is the sum of the counts in both controls and cases.

‡Note that the *P* values for the Fisher’s exact test are not corrected for multiple testing because the cluster2haps program is *only* used to identify the haplotypes involved in the haplotype clusters that are already found positive by Beagle *after* adjustment by permutations for multiple comparisons.

§The odds ratio (OR) is calculated for the haplotype being considered with regard to all other haplotypes as the reference. The 95% confidence intervals (CI) are also shown.

### Follow-up association study

The genotypes of the four SNPs that were followed up using Sample Set 2 were all in HWE. The SNPs rs220168 (S47, asymptotic *P* value (*P*_asym_) = 0.0074) and rs3819142 (S54, *P*_asym_ = 0.0021) were associated with extreme myopia defined by SE ≤ -10 D under a dominant genetic model (Table 4) while there was no significant finding with cases defined by SE ≤ -8 D or ≤ -12 D. Only the result of rs3819142 (S54) remained significant (*P*_emp_ = 0.0168) after correction for multiple comparisons (Table 4). The conclusion remained valid when sex and age were included as covariates because the *P* values only fluctuated slightly. Since these four SNPs did not show any significant association with high myopia at any of the SE thresholds tested in Sample Set 1 (see above and Table 2), we performed a combined analysis of these two sample sets for these four SNPs with cases defined by SE ≤ -10 D. The association signal for rs3819142 (S54) disappeared in the combined analysis (Table 4). Haplotype analysis did not reveal any significant finding either (data not shown). In other words, the overall results indicated that rs3819142 (S54) was not associated with high myopia.

**Table 4:** **Summary statistics of* UMODL1 *SNPs in follow-up study (cases defined as spherical equivalent ≤ −10.0 diopters)**

			**Allele†**		**Genotype counts (11/12/22)**		**Minor allele freq**		**Association test (best result)‡**		
SNP*		Location*	**1**	**2**	**Controls**	**Cases**	**Controls**	**Cases**	***P***_**asym**_	**Model**	***P***_**emp**_
**Follow-up study (Sample Set 2)**											
rs220168	S47	Intron	A	G	220/138/22	179/712/12	0.2395	0.1813	**0.0074**	Dominant	0.0568
rs220170	S48	Intron	C	T	228/120/22	178/70/15	0.2216	0.1901	0.1173	Dominant	0.5556
rs11911271	S49	Intron	T	C	205/157/27	138/117/14	0.2712	0.2695	0.7241	Dominant	0.9536
rs3819142	S54	Exon (ns)	A	C	215/149/20	182/76/10	0.2461	0.1791	**0.0021**	Dominant	**0.0168**
**Combined analysis (Sample Sets 1 and 2)**											
rs220168	S47	Intron	A	G	436/251/43	291/149/24	0.2308	0.2123	0.2883	Allelic	0.8884
rs220170	S48	Intron	C	T	439/243/41	294/145/27	0.2248	0.2135	0.4118	Dominant	0.9691
rs11911271	S49	Intron	T	C	380/305/54	256/183/32	0.2794	0.2622	0.3194	Dominant	0.9242
rs3819142	S54	Exon (ns)	A	C	420/279/39	294/154/23	0.2419	0.2123	0.0574	Dominant	0.3303

*SNPs are listed down the column in sequential order from the 5’ end to the 3’ end of the sense strand of the *UMODL1* gene. They are also designated as S47 to S49 and S52 as explained in the footnote to Table 2. The non-synonymous (ns) SNP rs3819142 (S54) is located in exon.

†1: major allele; 2: minor allele.

‡Single-marker analysis is performed with Plink. Asymptotic *P* values (*P*_asym_) are obtained by chi-square test, and empirical *P* values (*P*_emp_) obtained by 10,000 permutations for correcting multiple comparisons. The genetic models tested for each SNP are allelic, genotypic, additive (tested by trend test), dominant and recessive. Here, only the best result and the corresponding genetic model are shown for each SNP.

Interestingly, analysis of Sample Set 2 by Beagle also showed that the haplotype cluster 0.C allele of rs3819142 (S54) was associated with extreme myopia defined by SE ≤ −10D (Table 3). Upon exploring the cluster with the cluster2haps program, it was identified that the single marker rs3819142 (S54) gave the best *P* value (0.0055) with OR = 0.68 (Table 3) among other haplotypes. Single-marker analysis by Beagle produced a marginally significant result (*P*_emp_ = 0.0569) for the C allele of rs3819142 (S54). This implied that the association signal was not robust. Moreover, combined analysis of both sample sets for cases with SE ≤ −10D did not reveal this association signal. Consistent with the analysis by Plink, overall analysis by Beagle indicated that rs3819142 (S54) was not associated with high myopia. In summary, the putatively positive results of each sample set could not been replicated by the other sample set as well as the combined sample set.

### Association analysis of secondary phenotypes

In order to fully explore the ocular data and genotype data we had obtained, we conducted regression association analysis of secondary quantitative phenotypes (AXL, ACD, LT and CP) for Sample Set 1. No significant association was observed between these quantitative traits and any of the SNPs tested, except rs220120 (S23). Association of rs220120 (S23) with anterior chamber depth (ACD) was demonstrated for samples with cases defined by SE ≤ -8 D: *P*_asym_ = 0.001 and FDR-adjusted *P* = 0.046. The regression coefficient for the risk allele C of rs220120 (S23) was −0.066 (95% CI: -0.104 to −0.027). This means that each copy increase of the risk allele C *decreased* the anterior chamber depth by 0.066 mm on average. The association signal was stronger for a subset of Sample Set 1 with cases defined by SE ≤ −10D: *P*_asym_ = 0.0003 and FDR-adjusted *P* = 0.019 with the corresponding regression coefficient being −0.080 (95% CI: -0.124 to −0.036).

## Discussion

The *UMODL1* gene was first revealed as a new susceptibility gene for high myopia in a recent Japanese case–control study [8]. In particular, the SNP rs2839471 (S46) was found to be associated with high myopia (corrected *P* = 0.01 and OR = 1.68). Our study aimed at systematically investigating the *UMODL1* gene for association with high myopia in a Han Chinese population, and served to confirm or refute the original Japanese study. We genotyped and analyzed Sample Set 1 (Table 1) for 57 SNPs selected from the 86-kb region encompassing the *UMODL1* locus. Using five genetic models and cases defined by three different SE thresholds, single-marker analysis did not reveal any significant association between any single SNPs and high myopia after controlling for multiple comparisons. The conclusion remained the same no matter whether sex and age were included as covariates or not.

Haplotype-based analysis is more powerful in detecting association than single-marker analysis [27]. Different approaches of haplotype analysis offer different merits and disadvantages [22,28]. We performed haplotype analysis of our genetic data with three methods: LD-block-based method by Haploview, exhaustive variable-sized sliding window strategy by Plink, and localized haplotype clustering approach by Beagle. Of all these approaches, *only* Beagle identified a marginally significant (*P*_emp_ = 0.0763) haplotype cluster (0.G) for rs220168 (S47) for Sample Set 1 with cases defined by SE ≤ -12 D. Further analysis showed that three SNPs contributed to this haplotype association signal: rs220168 (S47), rs220170 (S48) and rs11911271 (S49) (Table 3). However, we failed to replicate this association signal with a second bigger sample set (Sample Set 2; Table 2) with all attempts (different SE thresholds for defining cases, and with or without adjustment for potential confounding by sex and age).

Instead, a non-synonymous SNP (rs3819142 or S54) showed significant association with high myopia for Sample Set 2 with cases defined by SE ≤ -10 D (Tables 3 and 4). This non-synonymous SNP was added in the follow-up study because it was in moderate LD (r^2^ = 0.6) with rs220170 (S48), which was one of the SNPs contributing to the positive haplotype cluster revealed in Sample Set 1. However, we failed to confirm the association signal of rs3819142 (S54) back in Sample Set 1 and in the combined sample set (Sets 1 and 2 combined), even with different strategies of haplotype analysis.

Overall, our results indicated that common polymorphisms in the *UMODL1* gene were not associated with susceptibility to high myopia in the Chinese population in Hong Kong. Power analysis strengthens the credibility of a genetic association study [5]. Therefore, power analysis is carried out using QUANTO (ver. 1.2.3) [29] for our study. We assume that the prevalence of high myopia is 0.05 in the Chinese population in Hong Kong [30], OR = 1.68 as obtained in the original Japanese study for the positive SNP rs2839471 (S46) and MAF = 0.2 (the cutoff for SNP selection for this study). We set the significance level at 0.00088 (=0.05/57) because 57 SNPs were analyzed for possible association with high myopia in the initial study. With a sample size of 356 controls and 356 cases, our initial study has ~80% power of detecting association under a log-additive genetic model. The power is almost 95% when the MAF is ~0.45 – the MAF of rs2839471 (S46) in the present study. Note that the power is expected to be less under other genetic models. This power analysis is valid only for single-marker analyses and a single phenotype (say, high myopia defined by a threshold of −8 D). We are also aware that the association analysis has been performed (1) for many more markers as defined by haplotypes and (2) many more phenotypes based on different thresholds for defining high myopia, and ocular quantitative traits (secondary phenotypes). The above-mentioned power analysis does not take into account these multiple markers and phenotypes. Although the permutation procedures of the analysis packages can handle both single markers and haplotypes together, they do not take into account the issues of multiple phenotypes. This will thus increase the false positive rates of the initial study. However, the main purpose of the initial study was to identify potential significant results so that they could be further examined in the follow-up study. The follow-up study used a larger sample size of 394 controls and 526 cases and examined 4 SNPs with 3 different definitions of high myopia and three different software packages. Assuming naively that the number of comparisons increases linearly with the number of multiple phenotypes examined by different packages, Quanto predicts that the power of the *follow-up study* is still well above 80%.

**Figure 1:**
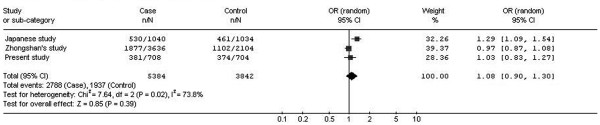
**Meta-analysis of case–control studies examining the association between high myopia and rs2839471 within* UMODL1 *.** Three studies are combined: the original Japanese study [8], a very recent Chinese study (Zhongshan’s study) [27], and the present study. The number (n) of the T allele of rs2839471 and the total number (N) of alleles (T and C) in the case group and the control group are shown in the second and third columns from the left. Note that rs2839471 (S46) was genotyped only in Sample Set 1 in the present study; there were 354 cases and 352 controls (see Table 2), and hence a total of 708 alleles in cases and 704 alleles in controls because 6 samples failed to be genotyped by iPLEX assays. The allelic odds ratios (OR) are shown diagrammatically in the middle, and numerically in the last column on the right. The contribution from each study is proportional to the size of the black square in the middle, and the exact weight is indicated in the second last column from the right. Overall, meta-analysis does not support the association between high myopia and rs2839471 (*P* = 0.39, DerSimonian and Laird method).

In our initial study, the SNP (rs2839471 or S46) originally positive in the Japanese study [8] was negative in all the analyses. The original Japanese study genotyped 39 SNPs for 520 controls and 520 cases with SE < −9.25 D [8], and 23 of these SNPs were within the same region examined by us. Of these 23 SNPs, 17 were also genotyped in our initial study. The remaining 6 SNPs were not tested in the present study because they did not satisfy the criteria of selecting tag SNPs – with an MAF <0.20 or not documented in the Han Chinese data of the HapMap Project. A very recent study *only* examined a single SNP (rs2839471 or S46) of the *UMODL1* gene for a very large group of unrelated Chinese subjects (n = 2,870) and reported *no association* between this SNP and moderate/high myopia (defined by different thresholds of spherical refraction at −4 D and −6 D) [31]. Therefore, we carry out meta-analysis to combine the data from all three studies for the SNP rs2839471 (S46) – the putatively positive SNP first identified by the Japanese study. For this purpose, we use the RevMan program (ver. 5.1.6) [32]. We note that different thresholds of SE or spherical refraction were used to define controls and cases with high myopia in these three studies. Therefore, we use a random-effects model for combining the data, as is supported by a significant test for heterogeneity (*P* = 0.02, Figure 1). Meta-analysis supports that there is no association between rs2839471 and high myopia: *P* = 0.39 (DerSimonian and Laird method for overall effect), and allelic OR = 1.08 (95% CI, 0.90 – 1.30) for allele T with reference to allele C (Figure 1).

Analysis of secondary phenotypes collected during case–control studies can provide valuable insights into biological pathways and help in identifying the association between genetic variants and phenotypes [25]. In the present study, no significant differences in AXL, CP and LT were found among different genotype groups in the case–control subjects. Instead, rs220120 (S23) showed association with ACD (*P*_asym_ = 0.001, FDR-adjusted *P* = 0.046). Intriguingly, this SNP is predicted to affect the binding of transcription factors (http://manticore.niehs.nih.gov/snpfunc.htm) and hence may affect the expression of UMODL1. However, the association signal may also be driven by a causal variant in strong LD with this SNP. If the association signal turns out to be genuine upon replication, it is worth examining the biological roles of the causal variant and UMODL1 in influencing ACD.

Refraction is determined by AXL, CP, LT and ACD. Of these, AXL is the primary determinant of high myopia, but not ACD [33]. Both AXL and ACD are highly heritable traits (heritability 0.67 for AXL and 0.78 for ACD) and are strongly correlated to each other [34]. While some of the genetic factors may be shared between AXL and ACD, others are unique to ACD [34,35]. Therefore, it is possible that the significant SNP for ACD is not associated with refraction. A genome-wide linkage study had identified 1p32 as a susceptibility locus for ACD [36]. Our results indicated that *UMODL1 might* be another candidate gene for ACD, but not AXL. Note that we did not take multiple secondary phenotypes into account while adjusting for multiple testing of SNPs. Therefore, the results of this *exploratory* study must be regarded as preliminary findings and need to be confirmed by additional studies.

## Conclusions

In conclusion, we systematically investigated the common polymorphisms of the *UMODL1* gene for association with high myopia by genotyping 59 SNPs in the initial study and 4 SNPs in the follow-up study. Both single-marker and haplotype analyses did not demonstrate any association with high myopia. However, exploratory association analysis of secondary phenotypes indicated the rs220120 was associated with ACD. We suggested that common polymorphisms of the *UMODL1* gene were unlikely to play an important role in the genetic susceptibility to high myopia in the Chinese population under study, and the role of *UMODL1* in ACD remained to be confirmed.

## Abbreviations

SNPs, Single nucleotide polymorphisms; *UMODL1*, The uromodulin-like 1 gene; AXL, Axial length; SE, Spherical equivalent; ACD, Anterior chamber depth; LT, Lens thickness; CP, Corneal power; MAF, Minor allele frequency; LD, Linkage disequilibrium; OR, Odds ratios; RFLP, Restriction fragment length polymorphism; DHPLC, Denaturing high performance liquid chromatography; PCR, Polymerase chain reaction; HWE, Hardy-Weinberg equilibrium; FDR, False discovery rate; SSLD, Solid spine of linkage disequilibrium; *P*_emp_, Empirical *P* values; *P*_asym_, Asymptotic *P* value.

## Competing interests

The authors declare that they have no competing interests.

## Authors’ contributions

MMZ carried out genotyping work, performed statistical analysis of single-marker and haplotype association analysis, and drafted the manuscript. MKH and YSG participated in the design of the study. DWHH carried out statistical analysis of secondary phenotypes. PWN recruited study subjects, and performed all the ocular examination. WYF processed blood samples and extracted DNA. SPY conceived, designed and coordinated the whole study, drafted and finalized the manuscript. All authors read and approved the final manuscript.

## Pre-publication history

The pre-publication history for this paper can be accessed here:

http://www.biomedcentral.com/1471-2350/13/64/prepub

## Supplementary Material

Additional file 1 **Primers for genotyping SNPs by MassARRAY iPLEX (Sequenom).** This table summarizes the primer sequences for genotyping SNPs by MassARRAY iPLEX (Sequenom). SNPs are arranged down the column in the sequential order from the 5’ end to the 3’ end of the *UMODL1* gene. Click here for file

Additional file 2 **PCR Primers and conditions for genotyping SNPs by RFLP and DHPLC.** This table summarizes the PCR primers and conditions for genotyping SNPs by RFLP and DHPLC. The restriction enzyme and the concentration of polyacrylamide gel used are indicated for each SNP being genotyped by RFLP. The primer for primer extension is shown for SNP genotyped by DHPLC. Some primers have a poly-T or poly-A tail at the 5’ end to enhance the difference in the size of restricted fragments for easy genotype calling. Click here for file

Additional file 3 **The gene structure and linkage disequilibrium (LD) pattern of the* UMODL1 *gene.** (A) The top panel shows the physical positions on chromosome 21, and the exon-intron organization for the isoforms of the * UMODL1 * gene at 21q22.3. (B) The bottom panel shows the distribution of 57 SNPs analyzed in this study and LD blocks as defined by the solid spine of LD algorithm of Haploview. The LD measures are indicated as r^2^ values. The intensities of the red colour indicate the magnitude with deep red being a value of 100% or 1 for the r^2^ value. Click here for file
