# Supplementary material for: Investigating the relationship between UMODL1 gene polymorphisms and high myopia: a case–control study in Chinese
Source: BMC Med Genet. 2012 Aug 2;13:64. doi: 10.1186/1471-2350-13-64 (PMC3489600; doi:10.1186/1471-2350-13-64)
Supplement: Additional file 3 — The gene structure and linkage disequilibrium (LD) pattern of the UMODL1 gene. (A) The top panel shows the physical positions on chromosome 21, and the exon-intron organization for the isoforms of the UMODL1 gene at 21q22.3. (B) The bottom panel shows the distribution of 57 SNPs analyzed in this study and LD blocks as defined by the solid spine of LD algorithm of Haploview. The LD measures are indicated as r2 values. The intensities of the red colour indicate the magnitude with deep red being a value of 100% or 1 for the r2 value. [file 1471-2350-13-64-S3.pdf]

Zhu MM, Yap MK, Ho DW, Fung WY, Ng PW, Gu YS, Yip SP.
The *UMODL1* gene as a susceptibility gene for high myopia: a case-control association study in Chinese

Additional file 3

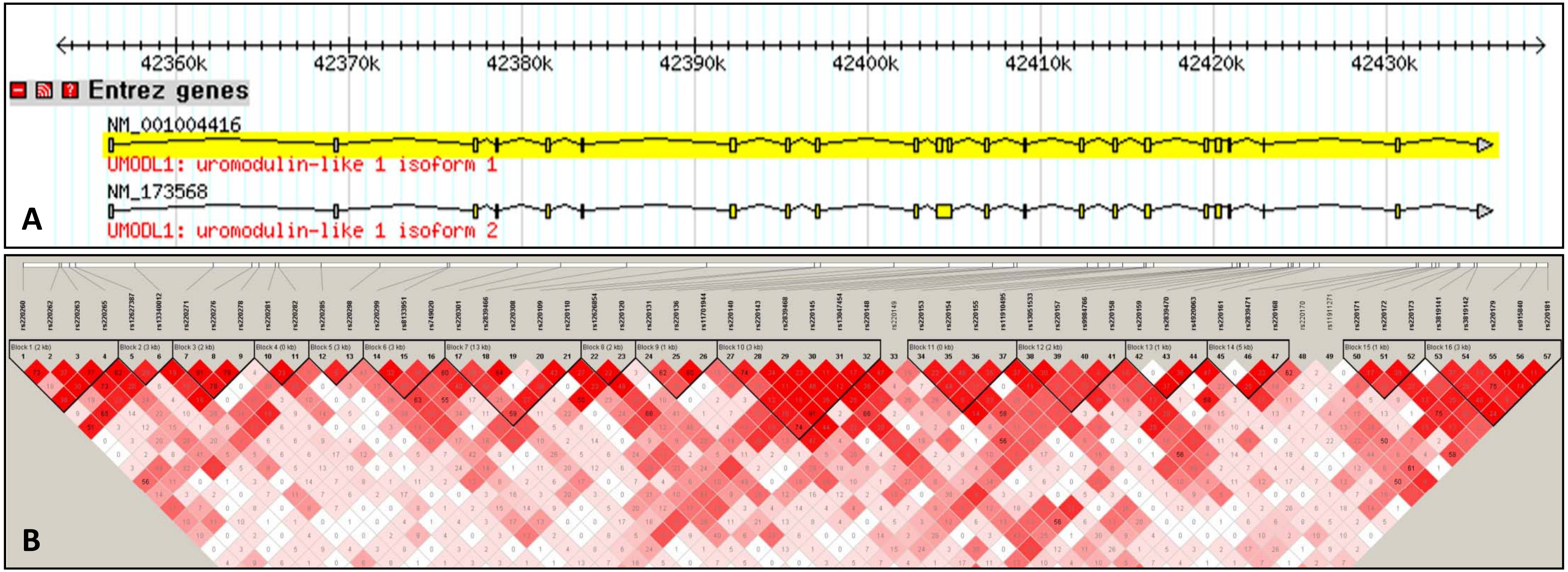

The gene structure and linkage disequilibrium (LD) pattern of the *UMODL1* gene.

(A) The top panel shows the physical positions on chromosome 21, and the exon-intron organization for the isoforms of the *UMODL1* gene at 21q22.3.

(B) The bottom panel shows the distribution of 57 SNPs analyzed in this study and LD blocks as defined by the solid spine of LD algorithm of Haploview. The LD measures are indicated as r^2 values. The intensities of the red color indicate the magnitude with deep red being a value of 100% or 1 for the r^2 value.
